# Formulation and evaluation of a novel herbal trio gel containing flax seed extract, carbopol and carboxymethyl cellulose

**DOI:** 10.6026/97320630019540

**Published:** 2023-05-31

**Authors:** P Swarna Meenakshi, Malaippan Sankari, Shanmugam Rajeshkumar

**Affiliations:** 1 Department of Periodontics, Saveetha Dental College and Hospitals, Saveetha Institute of Medical and Technical Sciences (SIMATS), Saveetha University, Chennai-600077, Tamil Nadu, India

**Keywords:** Flax seed extract, periodontitis, herbal intervention, SEM, FTIR, well-being, diseased

## Abstract

It is of interest to formulate and evaluate herbal trio gel containing brown flax seed extract, carbopol, and carboxymethyl cellulose and to assess the antimicrobial, anti-inflammatory activity along with quality analysis using SEM and FTIR. The brown flax seeds were grinded into a fine powder and supercritical fluid was prepared which was mixed with CMC and carbopol. The formulation was checked for antimicrobial, anti-inflammatory activity, surface characteristics with SEM and FTIR. The results revealed that the activity of the trio gel was efficacious in hampering the growth of black pigmented anaerobes. The highest zone of inhibition for novel herbal trio gel was recorded at 100 µL measuring 14mm and for the standard chlorhexidine gel it was recorded at 100 µL measuring 23mm in diameter. The results proved that the zone of inhibition of novel herbal trio gel had a decent difference to that of standard chlorhexidine gel. The anti-inflammatory activity showed significant activity at 20 µL which accounted for 53% for the novel herbal trio gel and for the standard diclofenac gel it showed highest activity at 20 µL which accounted for 60%. However, there was not much difference between the herbal trio gel and standard diclofenac gel. SEM observations revealed that the components used in formulation of this trio gel have been bonded well to each other and exhibited appreciable surface characteristics. The lattice of the trio gel has been very well exhibited in SEM analysis. FTIR revealed high peaks showing the different components present in the trio gel. Within the limitations of the study, the results of our study concluded that novel herbal trio gel containing *Linum usitatissimum* extract, sorbitol, and carboxymethyl cellulose could be an efficient economic primeval substitute that is non-toxic, natural, and structured for clinical application.

## Background:

Periodontitis is the primary elemental conflict that may serve as a nidus of infection and inflammation for the aetiology of a variety of other diseases. Periodontitis affects approximately 10-15% of adults worldwide, according to the World Health Organisation Global Oral Health Data Bank [1]. Periodontal disease is a chronic inflammatory periodontal disease characterised by periodontal ligament loss and destruction of surrounding alveolar bone in its advanced form [2]. Periodontitis is caused by a number of risk factors, including poor oral hygiene, diabetes, smoking, medications, cardiovascular events, hereditary and genetic factors, and stress [3]. However, age and heredity are risk factors that cannot be changed. Today's periodontitis treatment aims to reduce infection, resolve inflammation, and create a clinical condition compatible with periodontal health [4]. Periodontitis is typically treated initially in a non-surgical way. As a preventative measure, nonsurgical periodontal therapy is always an important tool in halting the progression of periodontal disease. Nonsurgical periodontal intervention may necessitate antibiotic coverage, but it is more often associated with an antibiotic resistance crisis, one of the world's most serious threats to health. In the oral cavity, several species of bacterial variants have been identified. It is accentuated that the complex interaction between the host response and the microbial species can progress to periodontal disease development [5][6]. Although non-surgical periodontal therapy results in clinical attachment gain and gingival margin recession due to inflammation resolution, residual pockets persist after treatment.

As an addition to nonsurgical periodontal therapy, systemic antibiotics given topically in the form of local drug delivery agents enhances the outcome by clearing residual pockets and limiting periodontal disease progression [6]. Systemic antibiotics aid the immune system by suppressing the target microbial species [7] [8]. The goal of utilising systemic antibiotics in conjunction with non-surgical periodontal therapy is to eliminate harmful bacteria while also establishing a healthy oral environment [8]. Resistance to certain antibiotics has been recorded, as has the evolution of resistant bacteria, both of which jeopardise antibiotics' beneficial action [9]. Cleansing systems include oral irrigation devices, dental floss, hydrogels, mouthwashes, chewing gum, and varnishes. Despite the fact that there are numerous cleansing agents available on the market, chlorhexidine gel has always been considered the gold standard and classical form of a cleansing agent that has been used exclusively for the past three decades, but not without its drawbacks such as taste disturbance, tooth discoloration, oral mucosal erosion, unilateral or bilateral parotid swelling [10][11]. Considering the limitations of the standard gel, alternative cleansing agents based on herbal products have been developed in the market. As a result, herbal therapies with therapeutic characteristics have grown in popularity in treating variety of disorders, and they are less prone to acquire resistance than antibiotics.

"Ayurveda" (Ayur - Life and Veda - Science), an Indian medicine system, has been utilised successfully to treat a variety of systemic diseases [12]. Since Vedic times, people have been using natural extracts to cure a variety of health problems all across the world. Flaxseed is a traditional awe-inspiring food that has been used in Ayurvedic therapy for thousands of years. Flaxseed (*Linum usitatissimum*), sometimes known as flax or linseed, is a plant in the Linaceae family that is high in omega-3 polyunsaturated fatty acids (PUFA), moisture, oil, calories, fibres, proteins, carbs, vitamins, and minerals [12]. It is also notable for its exceptional nutritional profile, which includes anti-bacterial, anti-inflammatory, anti-thrombotic, anti-cancer, and anti-arrhythmic properties [13] [14]. It has been shown to be a good source of omega 3 PUFA, which act as modulators of cell signalling, gene expression, and inflammatory processes [15][16]. Because of its high cysteine and methionine content, it is widely known for its hydroxyl radical scavenging and anti-oxidant activities [16,17]. Previous study has demonstrated that flax seed possesses anti-oxidant and anti-microbial effects against gram-positive pathogens. Flax seeds are offered in two varieties: brown flax seeds and golden flax seeds. Previous researches on golden flax seeds have also been conducted already. The main distinction between golden and brown flax seeds is their nutritional content, namely their fatty acid concentration. However, there is no data on brown flax seed extract as a local drug delivery agent and its qualitative analysis.

Cellulose is the most plentiful and least expensive natural polymer on the planet, with numerous appealing qualities such as biocompatibility, high moduli, low density, hydrophilicity, and high modification potential [18] [19]. Because of its water soluble nature, biocompatibility, and adaptability to various chemical changes, carboxymethyl cellulose (CMC), a cellulose derivative, has been widely employed to formulate local drug delivery agents.

Carbopol has sparked substantial interest as an excipient in a wide range of medicinal applications in recent decades [19]. Therefore, it is of interest to describe the formulation of a herbal trio gel containing flax seed extract, carbopol and carboxymethyl cellulose.

## Material and Methods:

## Fabrication of the trio gel:

Components used

[1] 200 g of flax seeds

[2] 0.5 g of carbopol

[3] 2.5 g of carboxymethyl cellulose

[4] 50 µL of peppermint oil

[5] 0.01 sodium benzoate

## Steps in preparation:

The trio gel was made by powdering freshly available brown flax seed. 2 g of brown flax seed powder was combined with 100 ml of distilled water and then weighed and blended. The solution was heated and then filtered. The supercritical fluid was boiled in a centrifuge to concentrate the filtered flax seed extract. Separately, 0.5 g of carboxymethyl cellulose was mixed with 10 ml of distilled water, and 0.5 g of carbopol was mixed with 10 ml of distilled water. Both solutions were combined and added to the concentrated brown flax seed extract. Sodium benzoate was added as a preservative and peppermint oil was added as a flavoring agent to the trio gel.

## Antimicrobial activity:

The antimicrobial activity of the trio gel was tested against black pigmented organisms isolated from plaque samples taken from periodontitis patients in paper points and transferred into a viable medium. The swab was then inoculated with black pigmented anaerobes. Mueller-Hinton agar was used to determine the inhibitory area (MHA). MHA was prepared and sterilized at 120 lbs for 45 min. The media was poured into the disinfected petri dishes and allowed to harden. The wells were cut with a well cutter, and black coloured organisms were swabbed from the plaque samples. The plates were filled with varying amounts of trio gel and incubated at 37°C for 24 hours. Following the incubation period, the zone of inhibition was assessed. The diameter of the inhibition zone, devoid of microbial growth, is clearly displayed.

## Anti-inflammatory activity:

## Albumin denaturation essay:

The anti-inflammatory effect of *Linum usitatissimum* (brown flax seed extract) was evaluated using the Mizushima and Kobayashi method, with a few changes [19]. 0.45 mL bovine serum albumin (1% aqueous solution) was added to 0.05 mL *Linum usitatissimum* gel at different concentrations (10L, 20L, 30L, 40L, 50L), and the pH of the trio gel was adjusted to 6.3 using a small amount of 1 N hydrochloric acid. These samples were kept at room temperature for 20 minutes before being heated in a water bath at 55°C for 30 minutes. A spectrophotometer was used to detect the absorbance at 660 nm after the samples has been cooled. Diclofenac sodium was utilized as a control. Dimethyl sulfoxide was utilized as a control.

Percentage of protein denaturation was calculated by the following equation:

% inhibition = (Absorbance of control - Absorbance of sample x 100)/ Absorbance of control

## SEM analysis:

The morphological characteristics of the trio gel were observed by scanning electron microscopy (SEM) [19]. At room temperature, the samples were initially coated with platinum using a sputter coater. SEM was used to evaluate the overall morphology of the triple gel after platinum plating it on a stub (JEOL JSM-IT800).

## FTIR analysis (Fourier transforms infrared analysis)

The trio gel was evaluated for the presence of organic components, including chemical bond, as well as organic content (protein, carbohydrate and lipid). FTIR helps to reveal the presence of various chemicals present in the trio gel. FTIR test was done in bruker's alpha II machine and the results were interpreted as percentage of transmittance at different wavelengths. More the percentage of transmittance, more the chemicals present in the trio gel has been cross linked to each other well indicating that the chemical bond between various chemicals is appreciable.

## Results:

## Antimicrobial activity

**Figure 1:**
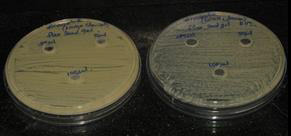
Antimicrobial activity of the novel trio gel at various concentrations against black pigmented anaerobes

**Figure 2:**
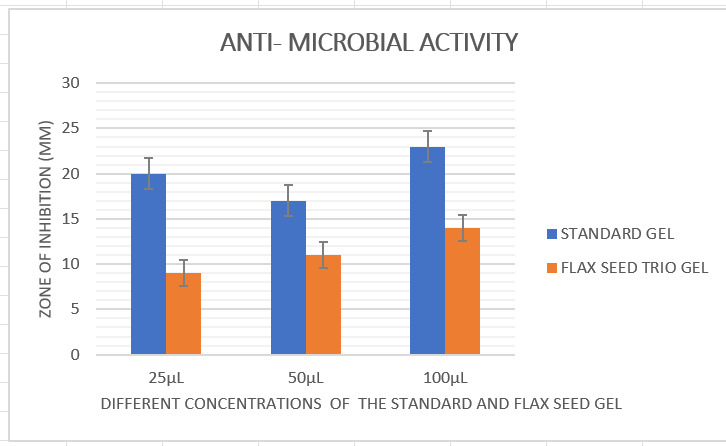
The below graph represents antimicrobial activity of novel trio gel and standard chlorhexidine gel at different concentrations against black pigmented organisms.

The results demonstrated that the trio gel's antibacterial action was effective in suppressing the growth of black pigmented anaerobes (Figure 1,Figure 2). This demonstrated the existence of a powerful zone of inhibition against black pigmented anaerobes. At different concentrations ie 25, 50, and 100 µL, black pigmented anaerobes had the highest zone of inhibition which is 9mm, 11mm, and 14 mm respectively. The standard chlorhexidine gel exhibited 20mm, 17mm, and 23mm respectively at 25,50, and 100 µL each. The commercial chlorhexidine gel had significant antimicrobial activity when compared to the novel trio gel. However, there is a reasonable amount of antimicrobial activity of this novel trio gel against black pigmented anaerobes, especially at 100 µL showing 14mm. The organisms were cultured in mueller hinton agar anaerobic jar.

## Anti-inflammatory activity:

**Figure 3:**
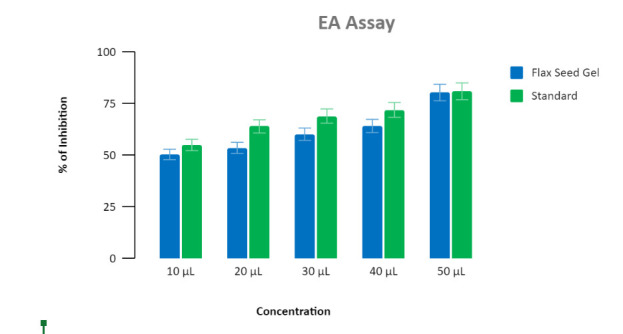
The below graph represents the anti-inflammatory activity showing the percentage of inhibition of novel trio gel and standard chlorhexidine gel at different concentrations.

The findings demonstrated that the percentage of inhibition was nearly identical to that of standard chlorhexidine gel. It was highest (53%) at 20 µL, followed by (60%) at 30 µL and least (71%) at 40 µL when compared to standard chlorhexidine gel. However, there was not much difference between both the gels. Our trio gel showed an acceptable anti- inflammatory activity compared to the standard chlorhexidine gel at 20 µL (Figure 3) (Table 1).

## SEM analysis:

**Figure 4:**
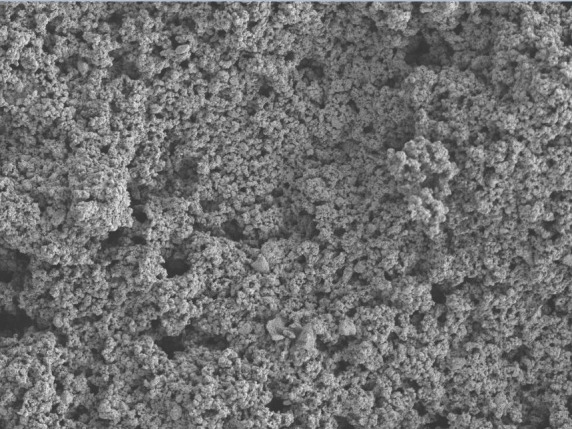
SEM analysis after 14 days, trio gel containing flax seed extract, CMC and carbopol has bonded well with each other.

SEM observations revealed that the trio gel had smooth surface characteristics. The lattice network of the novel herbal trio gel was tightly packed with each other. The particle size analysis showed that more than 90% of the formulated trio gel was within the size range of 41-65 µL in diameter. SEM analysis was tested at 1st and 2nd week after the gel has been formulated. It has been shown that the particles of the trio gel has been dispersed and bonded well to each other. This 14-day SEM analysis has revealed that mortification and the morphological changes of the trio gel were harmonious throughout this duration. No deterioration or wreckage of the trio gel components was noticed during the 14 days of delivery. SEM observations therefore revealed that the components used in formulation of this trio gel have been bonded well to each other and exhibited appreciable surface characteristics (Figure 4).

## FTIR analysis:

FTIR analysis revealed that there are peaks at 1000, 1500, and 1800 cm-1 respectively indicating that the trio gel is not a simple chemical by its structure. The FTIR revealed a sustained peak from 3000 cm-1 to 3300 cm-1 showing the presence of an aromatic structure. A narrow band is seen between 2500 and 3000 cm-1 revealing the presence of a C-C bond. There is a huge and sharp peak which was detected at about 1700 cm-1 indicating the presence of esters and a carboxyl group. Therefore, it can be concluded that this trio gel is a material that has an aromatic ring, and simple functional bonding (methyl). This confirms the presence of carboxyl group fatty acid, and polymer-containing aromatic structure. This is in good agreement with the chemical compound of Brown flax seed (*Linum usitatissimum*).

## Discussion:

We designed a substantiated release mechanism for *Linum usitatissimum* triple gel containing CMC and carbopol. Brown flax seeds are known for its nutritional and medicinal values and moreover it is known for reducing the inflammatory reactions in hypertension patients and cardiac patients [20,21]. A brown flax seed also maintain lipid profile and is rich in dietary fibers which are other form of vitamin E, thereby capable of binding with free radicals and executing standard anti-oxidant properties [22][23]. India is a country rich in medicinal herbs [24]. Carboxymethyl cellulose helps in enhancing the longevity of the trio gel and prevents it from degradation. It is known for its rheological properties and it deforms easily, starts to flow and maintains the fluid behavior [25]. Carbopol also exhibits appreciable shear strain and viscoelastic properties [26]. In an another study where gold flax seeds and brown flax seeds were compared in terms of lipid profile and blood pressure, brown flax seeds showed higher dietary fiber content when compared to gold flax seeds and also brown flax seeds had higher anti-oxidant activity than gold flax seeds [26]. Therefore the combination of brown flax seed extract, carboxy methyl cellulose and carbopol were prepared into a herbal trio gel. Herbal interventions are known for its nutritional and medicinal values [20]. Therefore goal of this research was to formulate a triple gel for local drug administration and evaluate its antimicrobial and anti-inflammatory activities, as well as quality analysis using SEM and FTIR. To the best of the author's knowledge, this is the first study to evaluate the surface features of a trio gel including brown flax seed extract, CMC, and carbopol.

The triple gel formulation demonstrated antibacterial activity which was almost in par with standard chlorhexidine gel. The triple gel revealed a sufficient zone of inhibition against the black pigmented anaerobes. The maximum zone of inhibition was detected at 100 µL, with a diameter of 14mm, among the various concentrations utilized for this formulation. When compared to standard gel, which had a zone of inhibition measuring 23mm, this was comparably satisfactory. By evaluating the zone of inhibition at varying concentrations, the study becomes more reliable. In the previous studies, antibacterial activity of gold flax seeds was checked against gram positive organisms like Streptococcus mutans which showed positive results where the number of bacterial colonies was reduced [27]. However this is the first study to evaluate the anti-microbial activity of brown flax seeds against black pigmented organisms which makes the study reliable. Although there have been a few earlier studies in which antimicrobial activity was tested, it was not examined at different concentrations, which prompted us to test the antimicrobial activity at different concentrations and reach a more reliable and acceptable result. In the current investigation, antibacterial activity was tested at various concentrations in order to get a more accurate conclusion about using this formulation as a local drug delivery system for periodontitis patients.

In the current study, anti- inflammatory activity was performed in bovine serum albumin which showed satisfactory percentage of inhibition and was in almost par with standard chlorhexidine gel. It was highest at 20 µL showing 53% of a zone of inhibition showing not much difference with standard gel which showed 60% at 20 µL. In another study, it has been proved that adequate dietary intake of omega-3 polyunsaturated fatty acids (n-3 PUFAs) increases tissue concentrations of fatty acids that help in subsiding inflammation. The anti- inflammatory activity of brown flax seed extract proved it to be an efficient formulation and hence can be used in patients as a local drug delivery agent in periodontitis patients which helps in reducing periodontal inflammation. In another study where the inflammatory response was compared between gold and brown flax seeds, brown flax seeds showed superior anti-inflammatory activity [29].

SEM was used in the current study to evaluate surface characteristics. According to the author's best knowledge, this is the first study to assess the qualitative properties of the herbal trio gel. SEM studies revealed that the surface of the herbal trio gel was smooth and uniform during the deterioration process. The herbal trio gel had a sustained release for 14 days. Previous research has shown that the optimal diameter of particles for intra-articular injection in rats is 35-105 µm, with no adverse effects. SEM revealed that the particle size was 35-71 µm in diameter, indicating that the herbal trio gel components may not be detrimental to the gingiva and may be suitable for distribution as a local pharmacological agent. As a result, cell migration is enhanced, leading to the resolution of the inflammation. SEM analysis further demonstrated that the emission was consistent and sustained for 14 days. During the 14-day release period, no aggregation or breakdown of chemicals in the herbal trio gel was detected.

The FTIR test revealed the existence of aromatic structure, carboxyl groups, and carbon bonds in the current study, indicating the presence of fatty acids and polymers. This FTIR revealed the existence of organic chemicals that are more beneficial to the gingiva and less toxic to it. As a result, this herbal trio gel is an important source of local drug delivery agents. There have been no previous studies that have assessed the quality of the innovative herbal trio gel. The intensity of cross linking among the various chemical compounds present in the herbal trio gel is also shown by FTIR. Because double covalent bonds constitute the strongest chain of cross linking, the components in the trio gel are well-packed with each other, clearly demonstrating the presence of omega-3 fatty acids.

Furthermore, CMC works as a polymer surface binder and was used in this study to boost the wettability of the herbal triple gel. Another study found that CMC-based hydrogels, such as CMC-PVA hydrogel, can be utilized in facial masks to improve moisture or water retention capacity [28]. This adds authenticity to the study since water retention capacity rises and degradation reduces, hence improving nourishment to the tissues. Non-surgical therapy requires additional adjuncts to improve outcomes and ensure effective plaque control[29]. Non-surgical therapy is usually an important component of overall treatment success. As a result, the prepared herbal trio gel can be employed as an addition to non-surgical therapy as well as a local drug delivery agent in periodontitis patients.

## Conclusion:

We were able to successfully formulate a novel herbal trio gel containing flax seed extract. Our study showed no significant difference between the novel brown flax seed extract herbal trio gel and standard chlorhexidine gel. Novel herbal trio gel showed a similar, comparable anti-inflammatory effect to standard chlorhexidine gel. The formulated trio gel is cost-effective and non- toxic, and easy to prepare. However cytotoxicity tests needs to be done in future studies for further use in patients.

## Figures and Tables

**Table 1:** Anti- inflammatory activity of novel trio gel and standard chlorhexidine gel at different concentrations

**Concentrations**	**Standard**	**Trio gel**
10µL	51	49
20µL	60	53
30µL	74	60
40µL	75	71
50µL	79	76
